# Somatosensory Misrepresentation Associated with Chronic Pain: Spatiotemporal Correlates of Sensory Perception in a Patient following a Complex Regional Pain Syndrome Spread

**DOI:** 10.3389/fneur.2017.00142

**Published:** 2017-04-10

**Authors:** Lars Büntjen, Jens-Max Hopf, Christian Merkel, Jürgen Voges, Stefan Knape, Hans-Jochen Heinze, Mircea Ariel Schoenfeld

**Affiliations:** ^1^Department of Stereotactic Neurosurgery, Otto-von-Guericke University, Magdeburg, Germany; ^2^Leibniz Institute for Neurobiology, Magdeburg, Germany; ^3^Department of Neurology, Otto-von-Guericke University, Magdeburg, Germany; ^4^Kliniken Schmieder Heidelberg and Lurija Institute, Allensbach, Germany

**Keywords:** complex regional pain syndrome, magnetencephalography, primary somatosensory cortex, cortical reorganization, pain spread

## Abstract

Chronic pain is suggested to be linked to reorganization processes in the sensorimotor cortex. In the current study, the somatosensory representation of the extremities was investigated in a patient with a complex regional pain syndrome (CRPS) that initially occurred in the right hand and arm and spread later into the left hand and right leg. After the spread, magnetoencephalographic recordings in conjunction with somatosensory stimulation revealed that the clinical symptoms were associated with major changes in the primary somatosensory representation. Tactile stimulation of body parts triggering CRPS-related pain elicited activity located in the left primary somatosensory region corresponding to the right hand representation, where the CRPS initially appeared. Solely the unaffected left foot was observed to have a regular S1 representation. The pain distribution pattern was matching the cortical somatosensory misrepresentation suggesting that cortical reorganization processes might contribute and possibly underlie the development and spread of the CRPS.

## Introduction

Complex regional pain syndrome (CRPS) is characterized by severe pain, dysfunction, and disability. The precise cause and pathophysiology are not known yet (1), and effective treatment options are limited and difficult (2). In the last decade, several reports emerged suggesting that the development, maintenance, and putative treatment of CRPS are associated with functional brain reorganization (3–6). In many studies, the primary somatosensory cortex (S1) showed changes with regard to the spatial organization but also to the magnitude of responses elicited by cutaneous stimulation [reviewed in Ref. (7)]. A recent meta-analysis revealed that the representation size of the affected hand in CRPS was smaller than that of healthy controls. One study showed a shorter distance between the representation of the lip and the hand in the affected S1 compared to the unaffected hemisphere (8). Mixed evidence has been presented with regard to the magnitude of activation in area S1. Hemodynamic studies showed smaller activation sizes and weaker activity in the CRPS-affected hemisphere (9–11) but also larger activations in the affected hemisphere (3, 4). There are also studies that found no differences in S1 activation between CRPS patients and controls (11, 12) in response to pain stimulation. Most studies employing electrophysiology (EEG) and magnetencephalography (MEG) also reported no differences between S1 responses in the affected and non-affected hemisphere (4, 13–15). However, some MEG studies did show a stronger response in S1 to stimulation of the affected compared to the unaffected hand (16). In comparison to healthy controls, no differences were reported so far (14, 16, 17). In summary, if at all, only small changes in amplitude and topographical representation can be expected in CRPS patient’s sensorimotor cortex when comparing affected and non-affected hemisphere responses to tactile stimulation.

Here, we report data from one patient with a CRPS that initially occurred in the right hand and arm. After failure of pharmacologic treatments, the patient received tonic epidural spinal cord stimulation (SCS) of the cervical spine creating paresthesias covering the area of pain. The patient did not experience pain attacks anymore and returned to his regular life. However, about 1 year later, the patient returned to the clinic and presented with a spread of the CRPS into the left hand and right leg associated with constant allodynia and piloerection. MEG recordings in conjunction with somatosensory tactile stimulation indicated that the evolvement of the clinical symptoms was associated with major changes in the primary somatosensory representation. Tactile stimulation of body parts triggering CRPS-related pain elicited activity located in the left S1 corresponding to the right-hand cortical representation, which matches the location where the CRPS initially appeared. Solely the unaffected left foot had a regular S1 representation.

## Methods

### Patient

The patient was 59 years old, and initially presented with very strong pain starting in the right hand and spreading within seconds to the forearm, upper arm, shoulder, and neck. Pain attacks were triggered by touch on the volar side of the right hand, later also by movement of the hand or forearm. In the occupational anamnesis, he reported an over a decade-long repetitive mechanical trauma to the right hand by using a heavy hammer during work. In addition, he had received hand surgery twice of the right and once of the left hand for carpal tunnel syndrome. The pain attacks aggravated in the context of a surgery of a disk-hernia in the segment C6/C7 and were reported to occur for about 2–5 min with a maximal strength of 10 points on a visual analog scale from 1 to 10 with 10 as the strongest possible pain. The pain was associated with loss of sensation, skin color change, and contraction of the arrector pili as vegetative reaction. The patient was diagnosed with CRPS, and due to the failure of analgesic medication (including steroids, anticonvulsants, antidepressants, and even opioids), he was implanted with an SCS electrode (octrode, the tip was placed at the level of the seventh cervical vertebra). The procedure was successful in that following stimulation no pain attack/vegetative reaction could be triggered/occurred anymore, while interruption of therapy lead to complete reoccurrence of symptoms within a few hours. However, after 1 year, the patient presented again with pain attacks now occurring in the left hand and right leg. The attacks had the same characteristics as the initial one in the right hand, were also associated with a concomitant vegetative reaction, and were/could be aggravated by touch, as now there was a constant pain component. Importantly, the pain was very different from other somatosensory deficits associated with his sensorimotor polyneuropathy caused by a diabetes type II. After extending SCS coverage, allodynia and autonomic disturbances subsided.

### Stimuli

Somatosensory tactile stimulation was applied in separate sessions to the volar side of the left and right hands and to the dorsum of the feet between the second and third metatarsal bone with a frequency of 0.5 Hz. The stimuli were delivered using a custom made pneumatic device *via* plastic tubes carrying compressed air to thin synthetic membranes taped to the skin. The pressure of the pneumatic devices was adjusted to have equal intensity in order to produce a robust sensory stimulation. The delay between triggering the air pressure and the delivery at the plastic membrane was 45 ms due to the length of the plastic tubes. These 45 ms are subtracted from the timeline, in that time point 0 is the time of the delivery of the stimulus at the membrane in contact with the skin. The tactile stimulation is often used in clinical context and typically elicits responses in the S1 with maxima at around 25 ms for dorsal hand and 45 ms for dorsal foot tactile stimulation. Special care was given to choose an intensity that did not trigger pain attacks. The intensity of the tactile stimulus was slowly increased to the maximum tolerable level of the right hand. Based on his experience, the patient gave feedback in order to avoid triggering a pain a pain attack. This intensity level was then employed for all extremities. Each extremity-specific run was about 6 min long and contained 180 stimuli. The experimental session in the MEG consisted of four runs and took about 50 min including preparation. Ten hours before the recordings, the spinal stimulation was turned off.

### ERMF Recordings

Event-related magnetic fields were recorded using a Magnes 3600 WH (4D neuroimaging, San Diego, CA, USA) whole-head system with 249 magnetometers. Recording bandpass was DC-50 Hz with a sampling rate of 254 Hz. The data were segmented into epochs from −25 to 175 ms time locked to the stimulus delivery, filtered offline with a bandpass from 0.1 to 30 Hz filter and baseline corrected for the offset between −25 and 0 ms. Artifact rejection was performed offline by removing epochs with peak-to-peak amplitudes exceeding a threshold of 3.0 × 10^−12^ T. This procedure also rejected epochs with eye and head movements, as well as artifacts caused by the implanted spinal cord stimulator and electrode, which was ensured by visual inspection. Rejection rates varied between 36 and 40% of the trials leaving a minimum number of at least 108 trials per condition. The individual head shape of the patient was co-registered with the sensor coordinate system by digitizing (Polhemus 3-D Space Fastrak) skull landmarks (nasion, left and right preauricular points) and determining their locations relative to sensor positions using signals from five distributed head coils. These landmarks enabled co-registration of ERF activity with the anatomical brain model.

### Event-Related Magnetic Fields and Source Analysis

Separate artifact-free averages were formed for each of the four conditions (left and right hand, left and right foot). The left and right hand averages exhibited clear amplitude peaks around 25 ms poststimulus corresponding to the early cortical components generated in the S1 typically observed in the EEG (N25) (18). In the conditions with foot stimulation, the averages exhibited clear first amplitude peaks around 45 ms. For the conditions with hand stimulation, source analyses were performed in the time range around the peak (20– 30 ms). For the foot stimulation conditions, the analyses were carried out in the time range 40–65 ms. All source analyses were performed using multimodal neuroimaging software (Curry 7.0, Compumedics/Neuroscan Inc.). Source modeling was performed using minimum L2-norm estimates in a realistic boundary element model of the head derived from an MRI of a subject with similar head size as the patient since it was impossible to obtain an MRI of the patient’s brain due to contraindications related to the implanted spinal stimulation device. The source activity was thresholded at 70% of the maximum source strength within the time range. The procedures follow previous work of our group (19, 20).

## Results

### Pneumatic Somatosensory Tactile Stimulation of the Right Hand

**Figure 1:**
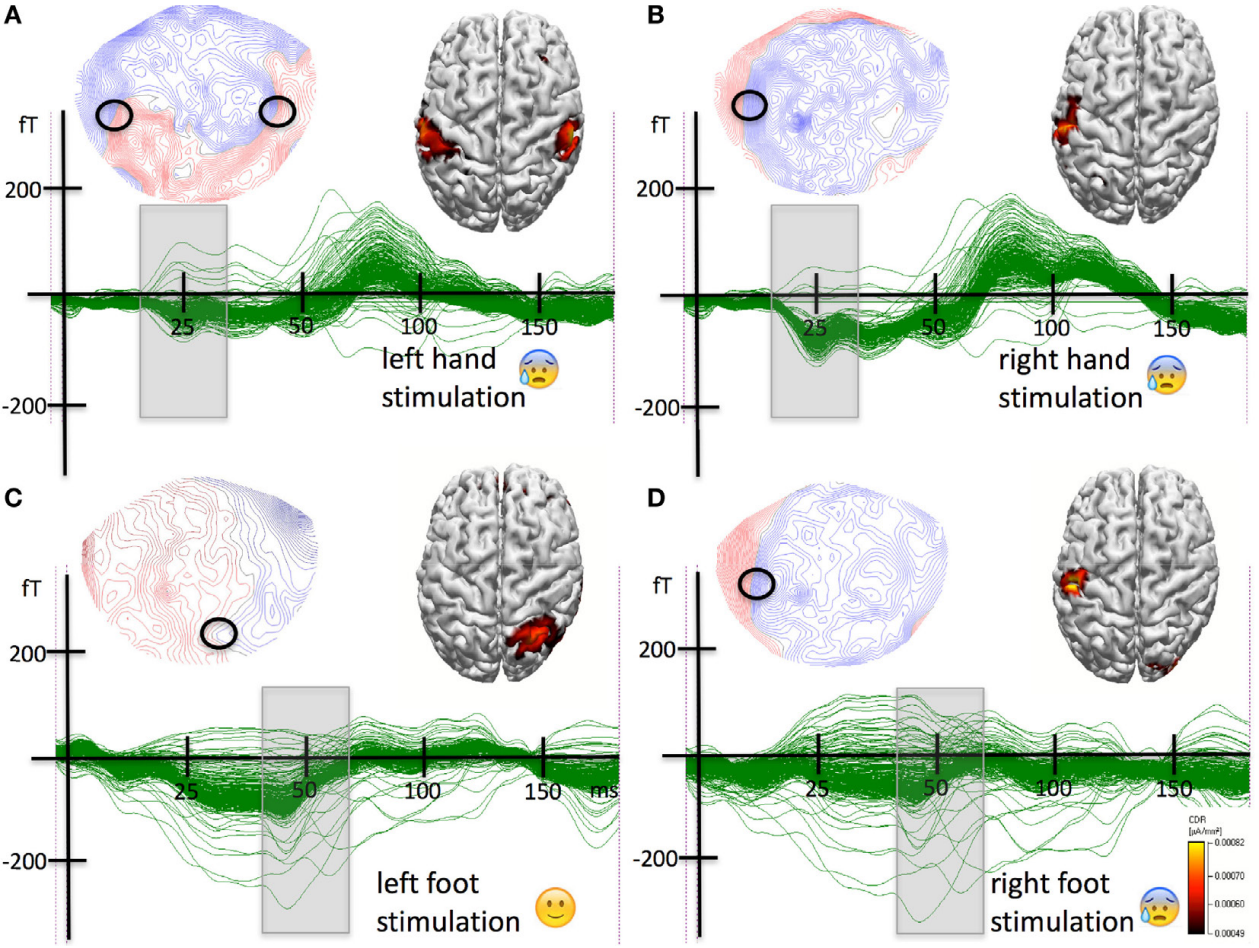
**(A)** Results for the tactile stimulation of the left hand. Strong stimulation of the left hand typically triggered strong pain. A butterfly plot of the magnetoencephalographic (MEG) waveforms elicited by pneumatic tactile somatosensory stimulation to the volar side of the left hand along with the event-related magnetic field topographical distribution for the first peak of activity (20–30 ms poststimulus) and the results from the source analysis are shown. The field has a quadrupolar distribution with polarity reversals over the ipsi- and contralateral hemisphere (indicated by the black circles). The sources were localized to the left and to the right primary somatosensory cortex matching the representation of the hand knobs. **(B)** Results for the tactile stimulation of the right hand. Typically, stimulation of the right hand triggered strong pain. Initially, the pain syndrome was restricted to the right hand and arm. A butterfly plot of the MEG waveforms elicited by pneumatic tactile somatosensory stimulation to the volar side of the hand along with the event-related magnetic field topographical distribution for the first peak of activity (20–30 ms poststimulus) and the results from the source analysis are shown. The field has a clear dipolar distribution over the contralateral left hemisphere. The circle indicates the potential reversal underlying the source. The sources were localized to the left primary somatosensory cortex, more precisely to the left hand knob. **(C)** Results for the tactile stimulation of the left foot. The stimulation did not trigger any pain. A butterfly plot of the MEG waveforms elicited by pneumatic tactile somatosensory stimulation to the to the dorsal left foot between the second and third metatarsal bone is shown along with the event-related magnetic field topographical distribution for the first peak of activity (40–65 ms poststimulus). The field has a dipolar distribution with polarity reversals over the contralateral right hemisphere (indicated by the black circle). The sources were localized to the right postcentral gyrus corresponding to the right primary somatosensory cortex representation of the foot. **(D)** Results for the tactile stimulation of the right foot. Strong stimulation typically triggered pain. The left bottom part of the figure shows a butterfly plot of the MEG waveforms elicited by pneumatic tactile somatosensory stimulation to the dorsal right foot between the second and third metatarsal bone. Above the butterfly plot, the event-related magnetic field topographical distribution for the first peak of activity (40–65 ms poststimulus) is shown. The field has a dipolar distribution with polarity reversals over the ipsilateral left hemisphere. The right bottom part of the figure shows the results of the source analysis of the magnetic field distribution in the corresponding time range. The sources were localized to the left postcentral gyrus corresponding to the primary somatosensory cortex representation of the hand knob.

The stimulation elicited robust evoked magnetic fields with a first amplitude peak at around 25 ms poststimulus (see Figure 1A), which is similar to observations using the same stimulation parameters in normal subjects. In the time range around this peak (20–30 ms), a clear bipolar topographic distribution was observed over the left hemisphere. Source analyses localized the generators of this magnetic field distribution in the left somatosensory and partially motor cortex. This region corresponded to the postcentral gyrus and the analogous part in the precentral gyrus so-called “hand knob” (21). Importantly, the neural representation was unilateral and contralateral to the tactile stimulation site.

### Pneumatic Somatosensory Tactile Stimulation of the Left Hand

The stimulation elicited an evoked magnetic field with a first amplitude peak at around 25 ms poststimulus (Figure 1B). The magnetic field in the time range around the peak had a quadrupolar distribution with maxima/minima combinations over the left and right hemispheres. The source analyses revealed two estimates of neural activity located in the postcentral/precentral gyrus around the hand knob in the left and in the right hemisphere. In contrast to the tactile stimulation of the right hand, the neural representation revealed by left hand stimulation was bilateral and included the contralateral but also the ipsilateral somatosensory and motor cortex.

### Pneumatic Somatosensory Tactile Stimulation of the Right Foot

The stimulation elicited evoked magnetic fields with a first amplitude peak at around 55 ms poststimulus (Figure 1C). In this time range around the peak (40–65 ms), a clear bipolar topographic distribution was observed over the left hemisphere. Source analyses localized the generators of this magnetic field distribution in the left somatosensory and partly motor cortex in the postcentral/precentral gyrus around the hand knob. The source was strictly contralateral to the stimulation site.

### Pneumatic Somatosensory Tactile Stimulation of the Left Foot

The stimulation elicited evoked magnetic fields with a first amplitude peak at around 55 ms poststimulus (see Figure 1D). In the time range around the peak (40–65 ms), a clear bipolar topographic distribution was observed over the central-posterior right hemisphere. Source analyses localized the generators of this magnetic field distribution in the right somatosensory cortex in the postcentral gyrus next to the midline. The unilateral source was strictly contralateral to the stimulation site.

**Figure 2:**
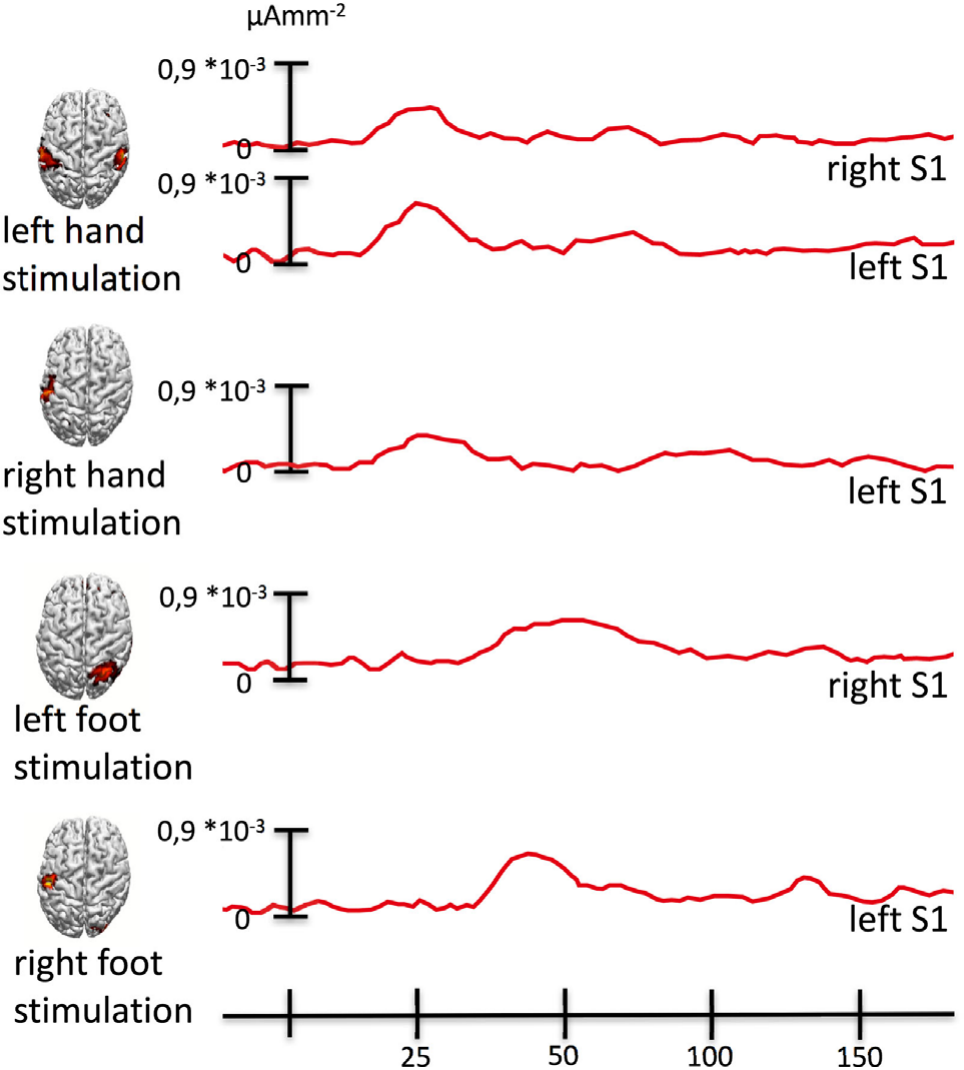
**Source activity waveforms**. The figure shows source activity (mean activity in a sphere of 5 mm radius around the maximum) across experimental conditions. Whenever the tactile stimulation was delivered to an extremity to which strong stimulation would have triggered pain, we also observed sources of the first peak of activity to be located in the left hand knob of the left primary somatosensory cortex corresponding to the representation of the right hand, in which the pain initially (before the spread) occurred.

In summary, the earliest responses to tactile stimulation of the right hand were generated in the contralateral left hand areas of sensorimotor cortex. The somatosensory representation of the left hand was located in the contralateral hand areas but also in the ipsilateral hand areas of the left hemisphere. The responses to tactile stimulation of the right foot were generated in the hand areas of the sensorimotor cortex of the contralateral left hemisphere. The somatosensory representation of the left foot revealed by tactile stimulation was located strictly in the somatosensory cortex of the contralateral right hemisphere next to the midline in the region corresponding to the foot area. Note that the hand area of the somatomotor cortex of the left hemisphere was always part of the neural representations of body regions associated with the triggering of pain (left and right hand and right foot) regardless of the body side (Figure 2).

## Discussion

Complex regional pain syndrome typically starts in one part of the body and can spread to the other extremities. Contralateral spread is twice as likely as ipsilateral, and diagonal spread is rather rare (17). There is a strong body of evidence showing changed functional organization of the primary sensory cortex associated with CRPS [reviewed in Ref. (7)]. In sum, most studies converge on smaller representations of the body parts associated with CRPS and to some extent on modulations of the response amplitudes in EEG/MEG measures or changes in the magnitude and size of activated clusters in hemodynamic measures. Interestingly, a recent study (22) employed MEG in conjunction with somatosensory stimulation and showed a maladaptive reorganization of the representations of fingers I and V associated with CRPS that was restored through SCS. Less is known on the spreading of CRPS although it seems to be a quite common phenomenon. About 48% of the patients experience a spread of the CRPS over a 6-year period. In about one-half of these, it went to the contralateral limb. Only in 15%, it spread in a diagonal pattern (17). The neural mechanisms of the spread are yet unclear.

Here, we report data from a patient who initially presented with a CRPS in the right upper extremity. A mechanical trauma to the hand was present in his anamnesis, and he fulfilled the Budapest criteria (23) for the diagnosis. Exerting pressure on the volar side of the hand could reliably trigger pain attacks of utmost severity. After failure of analgesic drug therapy, the patient was treated with SCS covering the entire right upper extremity. This was initially successful in inhibiting the pain attacks. However, within 1 year, the CRPS spread to the contralateral extremity and to the ipsilateral leg. We investigated the earliest event-related magnetic field correlates of cortical processing of tactile stimulation of the limbs. Right-hand tactile stimulation (Figure 1B) elicited a left S1 response from the hand knob (21). The tactile stimulation of the newly affected left hand elicited activity in the contralateral hand knob but surprisingly also in the left one (see Figure 1A) corresponding to the representation of the right hand where the CPRS initially came up. Even more surprisingly, the tactile stimulation of the newly affected right foot elicited early activity exclusively in the left hand knob (see Figure 1D). Tactile stimulation of the unaffected left foot elicited a response corresponding to the known location of the foot representation on the somatosensory homunculus (Figure 1C). In short, affected body part stimulation was associated with a cortical response originating from the left hand knob, the neural representation of the first affected body part (Figures 1 and 2). This major misrepresentation with strong contributions of the hand area of the left S1 in the responses to CRPS-affected limbs indicates a deeper involvement of S1 in comparison to previous reports [reviewed in Ref. (7)]. The main difference between this and previous report is that here we investigated the neural correlates after the spread of the CRPS. The findings suggest a key role of S1 in the spread, especially of the representation of the initially affected limb.

Recent work [reviewed in Ref. (24)] suggests an early intimate relationship between S1 across hemispheres especially for the hands that challenges the classical textbook knowledge of unilateral tactile representation. Tactile information from the body side appears to reach early ipsilateral S1 *via* transcallosal connections supporting bilateral integration in early stages of tactile processing. Importantly, these processes are dependent of task demands. It is thus conceivable that CRPS might also trigger such processes, which would provide a theoretical framework for the spreading pattern (since the most common pattern is the spread across homologous extremities). In the same vein, allodynia and its spreading are associated with bilateral activation of the thalamus (25), thus providing a rationale for bilateral activation of the cortex.

Changes in the functional organization of the sensorimotor cortex have also been observed in other conditions causing disability such as cerebral palsy (26). Furthermore, the interactions within the sensorimotor network are also disturbed (27). A recent study employed a multimodal neuroimaging approach and showed changes in somatotopy in cerebral palsy children (28). The reorganization in the sensorimotor network of children with CP was interpreted as a result of diminished thalamocortical projections. This is well in line with the current findings in chronic pain, in which a similar mechanism could have driven the observed changed somatosensory representation of pain-associated limbs.

There are several limitations to the present study. We did not have the chance to map the S1 representation of the limbs in the initial phase of the CRPS when only the right limb was affected. It is, however, reasonable to assume that before the CRPS, the patient’s representation of the limbs in S1 was contralateral. Based on the literature (22), in the initial phase of the CRPS, our patient would not have had these major changes in the cortical representation of the limbs found here. In the same vein, the reported changes closely match the clinical symptoms after the spread. It is likely that they are related to the spread and not to the initial representation but the current data neither show nor prove it. More patients need to be studied with the same approach in order to draw stronger inference.

In sum, the present data suggest an important role of the S1 in CRPS. Current research in this field needs to address the phenomenon of spreading in order to elucidate the underlying neural mechanisms. Given that almost one-half of the CRPS patients experience a spread, a wider coverage of the spinal cord electrode could be helpful and could help avoiding a second surgery of the spine.

## Ethics Statement

The subject gave written informed consent in accordance with the Declaration of Helsinki. The protocol was approved by the ethics committee of the medical faculty of the University of Magdeburg.

## Author Contributions

LB, J-MH, CM, JV, H-JH, and MAS planned and designed research. SK and MAS performed measurements. LB, CM and MAS analyzed data. LB and MAS wrote the manuscript.

## Conflict of Interest Statement

The authors declare that the research was conducted in the absence of any commercial or financial relationships that could be construed as a potential conflict of interest.
